# Tracking Macular Sensitivity and Inner Retinal Thickness in Long-Term Type 1 Diabetes: A Five-Year Prospective Examination in Patients without Diabetic Retinopathy

**DOI:** 10.3390/life14091152

**Published:** 2024-09-12

**Authors:** Guisela Fernández-Espinosa, Elvira Orduna-Hospital, María Sopeña-Pinilla, Marta Arias-Álvarez, Ana Boned-Murillo, María Dolores Díaz-Barreda, Ana Sánchez-Cano, Isabel Pinilla

**Affiliations:** 1 Aragon Institute for Health Research (IIS Aragon), 50009 Zaragoza, Spain; guisela.fernandez3@gmail.com (G.F.-E.); mariasopenapinilla@gmail.com (M.S.-P.); martariasalvarez7@gmail.com (M.A.-Á.); anabomu@hotmail.com (A.B.-M.); lodiba92@gmail.com (M.D.D.-B.); anaisa@unizar.es (A.S.-C.); 2 Department of Applied Physics, University of Zaragoza, 50009 Zaragoza, Spain; 3 Department of Ophthalmology, Miguel Servet University Hospital, 50009 Zaragoza, Spain; 4 Department of Neurophysiology, Lozano Blesa University Hospital, 50009 Zaragoza, Spain; 5 Ophthalmology Mediterranean Foundation (FOM), 46015 Valencia, Spain; 6 Department of Ophthalmology, Virgen de la Luz Hospital, 16002 Cuenca, Spain; 7 Department of Ophthalmology, Lozano Blesa University Hospital, 50009 Zaragoza, Spain; 8 Department of Surgery, University of Zaragoza, 50009 Zaragoza, Spain

**Keywords:** type 1 diabetes mellitus, microperimetry, spectral domain optical coherence tomography (SD-OCT), retinal thickness, macular sensitivity

## Abstract

The aim of the study is to compare macular sensitivity and retinal thickness in patients with long-term type 1 diabetes mellitus (DM1) without diabetic retinopathy (DR) after 5 years of follow-up. Thirty-two eyes from 32 long-term DM1 patients without DR were included. All participants underwent a complete ophthalmological examination, including microperimetry and spectral domain optical coherence tomography (SD-OCT). The data were compared with results from 5 years prior. The mean age of the DM1 patients was 43.19 ± 10.17 years, with a mean disease duration of 29.84 ± 8.98 years and good glycemic control. In 2023, patients exhibited a significantly worse best corrected visual acuity (BCVA) compared to 2018 (*p* < 0.001). DM1 patients did not show statistically significant changes in macular sensitivity over the 5-year follow-up period. Macular integrity showed significant differences between the two time points (*p* = 0.045). Retinal thickness showed significant differences, particularly in inner retinal layers (IRL) across most of the ETDRS areas. Long-term DM1 patients without DR lesions showed worsened macular integrity and a lower BCVA in 2023. Additionally, they displayed significant alterations in retinal thicknesses, especially in the IRL, between 2018 and 2023. These findings suggest that even in the absence of visible DR, long-term DM1 patients may experience subclinical retinal changes and functional deterioration over time, highlighting the importance of regular monitoring for the early detection and management of potential complications.

## 1. Introduction

Diabetes mellitus (DM) is a heterogeneous metabolic disease characterized by chronic hyperglycemia [1]. The prevalence of DM is projected to increase, with an estimated 629 million individuals affected by 2045 [2]. Diabetic retinopathy (DR), a primary microvascular complication of DM, is the leading global cause of blindness in working-age individuals [3].

DM represents a notably specific neurovascular complication, mainly associated with the duration of diabetes and glycemic control [4]. Numerous studies have demonstrated functional changes occurring before the onset of vascular signs [5,6]. Retinal neurodegeneration affects both function and retinal structure, leading to a decrease in retinal sensitivity (RS) or alterations in the thickness of the inner retinal layers (IRL) [5,7,8].

The daily practice for the DR diagnosing and managing relies on the assessment of visual acuity (VA) and multimodal imaging, which includes examining macular morphology and retinal layer thicknesses through optical coherence tomography (OCT) [9]. Despite some long-term DM patients being protected from developing DR, they may still experience functional and anatomical changes [10,11,12]. Even in the absence of visible anatomical retinal changes, visual function could be compromised [13,14].

Microperimetry is a rapid and non-invasive test that measures RS. It offers several advantages over traditional perimetry, including increased speed, greater sensitivity, and the ability to monitor RS consistently in subsequent examinations by measuring at the same location thanks to the integrated eye-tracker [15]. The test determines the lowest light intensity that patients can detect when light spots stimulate specific macular areas while also evaluating gaze fixation stability [16]. Light stimulus intensity is measured in decibels (dB), with a higher RS score indicating the detection of a dimmer stimulus [17,18].

RS may be compromised even in DM patients without DR signs with preserved VA. Microperimetry proves to be a valuable technique, as it can reveal functional changes before the onset of vascular manifestations [8,19,20].

OCT is another non-invasive test that facilitates the quantification of retinal layer thicknesses and volume changes [21]. In order to assess neurodegeneration in DM patients, we investigated alterations in macular sensitivity assessed through microperimetry and changes in the thicknesses of the inner retina, the outer retina, and the retinal nerve fiber layer (RNFL) using OCT in long-term type 1 DM (DM1) patients who showed no signs of DR after a 5-year follow-up period.

## 2. Materials and Methods

### 2.1. Study Design

A cross-sectional study was conducted at the Ophthalmology Department of the Lozano Blesa University Hospital (Zaragoza, Spain). A total of 56 eyes from 56 DM1 patients were analyzed using the Macular Integrity Assessment system (MAIA) (Topcon Corporation, Tokyo, Japan) in 2018.

Patients underwent thorough fundus examinations and full-field retinography to confirm the absence of any DR sign by two different clinicians. In both 2018 and 2023, exclusion criteria for all participants included amblyopia or a best corrected VA (BCVA) less than 20/40 on the Snellen chart (0.3 in logarithm of the minimum resolution angle, LogMAR), refractive error exceeding 3.00 D of astigmatism or 5.50 diopters (D) of spherical equivalent (SE), intraocular pressure (IOP) above 20 mmHg, glaucoma with perimetric involvement or optic nerve atrophy, a history of any ocular or systemic disease affecting central vision, having developed any signs of DR, or inability to perform microperimetry (due to incomplete measurement, lack of fixation, or poor cooperation). Among these 56 eyes, 24 eyes were excluded due to various reasons: 11 eyes were lost to follow-up; 1 eye was diagnosed with glaucoma; 6 eyes developed DR, with 2 eyes requiring vitrectomy and another 2 eyes receiving photocoagulation; and 2 eyes had poor image quality due to cataracts. The final sample included 32 eyes of DM1 patients without signs of DR in 2023.

Approval for this study was obtained from the local ethics committee for Clinical Research of Aragon (CEICA PI17/0298 and PI23/063), and the evaluation was conducted following the principles of the Helsinki Declaration. Detailed consent forms were obtained from each subject.

### 2.2. Study Protocol

All subjects underwent a complete ophthalmological evaluation conducted by designated researchers to ensure consistency and minimize bias, which included the assessment of BCVA, expressed in LogMAR, using the ETDRS chart. Axial length (AL) was determined by the Aladdin KR-1 W Series optical biometry system (Topcon Corporation, Tokyo, Japan), and IOP was measured using Goldmann tonometry. The AL was calculated as the mean of 5 measurements and expressed in millimeters. Each test was conducted by the same observer throughout the study: OCT by M.S.-P., microperimetry by G.F.-E., and the previous ophthalmological tests by M.A.-A., A.B.-M., and M.D.D.-B., respectively. Fundus examination was performed by I.P. and M.S.-P. In addition to the ophthalmological evaluation, a thorough medical history was obtained, encompassing all aspects related to the patient’s disease. This included the time of diagnosis and other diabetes-related values, such as blood glycosylated hemoglobin (HbA1c), lipid levels, and renal function.

Microperimetry was performed to evaluate macular sensitivity and functional integrity. The MAIA microperimetry was conducted under scotopic conditions and without pupillary dilation. The MAIA is designed to identify any decrease in sensitivity compared to normal sensitivity related to age and pathological changes associated with retinal disorders.

The evaluated MAIA parameters included fixation loss, RS in 37 macular sensitivity points, macular integrity index, mean total threshold, fixation stability (P1 and P2, classified as stable (P1 > 75%), relatively unstable (P1 < 75% and P2 > 75%) or unstable (P2 < 75%)), bivariate contour ellipse area (BCEA) where the fixation points are contained, BCEA63 (minor ellipse), and BCEA95 (major ellipse).

**Figure 1:**
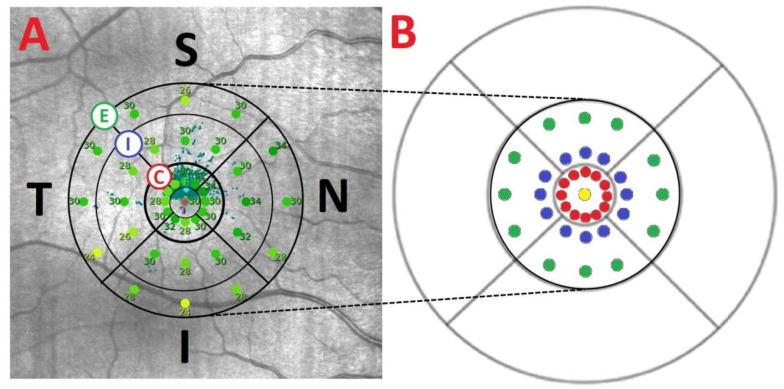
(**A**) Sensitivity points from a right eye measured by MAIA microperimeter with the 3 concentric rings (C: central, I: internal, and E: external). (**B**) The distribution of the 37 sensitivity points represented in an ETDRS grid: 1 in the center (yellow), 12 in the 1° radius (red), 12 in the 3° radius (blue), and 12 in the 5° radius (green). One degree is equivalent to a radius of 0.3 mm; 3° to a radius of 0.9 mm; and 5° to a circle with a 1.5 mm radius. The center point and the 1° sensitivity points (0.6 mm diameters) correspond to the ETDRS 1 mm diameter center ring, and the localized sensitivity points at 3 and 5° (diameters of 1.8 and 3 mm, respectively) to the 3 mm diameter parafoveal ring of the ETDRS grid. Abbreviations: S, superior; T, temporal; I, inferior; N, nasal.

The 37 macular sensitivity points examined by MAIA are distributed across a central point and 3 concentric rings (central, internal, and external) with 12 points in each ring. The points are positioned as follows: a central point, a central ring with a radius of 0.3 mm, an internal ring with a radius of 0.9 mm, and an external ring with a radius of 1.5 mm. We categorized the 37 sensitivity points into 14 MAIA areas (Figure 1). Each ring was divided into 4 areas (superior, temporal, inferior, nasal), central point area, and central global area.

Total retinal (TR) thickness, IRL, outer retinal layers (ORL), and RNFL were evaluated with the fast macula protocol using Spectralis spectral domain (SD)-OCT (Heidelberg Engineering, Inc., Heidelberg, Germany). Spectralis SD-OCT provides a circular macular grid analysis divided into nine sectorial thickness measurements in three concentric circles with diameters of 1, 3 (inner, parafoveal), and 6 (outer, perifoveal) mm, forming the 9 areas corresponding to the ETDRS grid. The quality of the scans was assessed, and poor-quality scans were excluded. Images were required to achieve at least 25 out of 40 dB.

**Figure 2:**
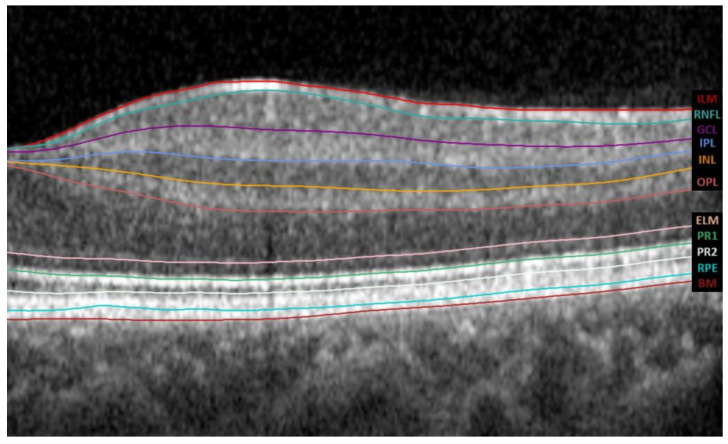
The tomographic profile obtained by Spectralis SD-OCT; the automatic segmentation was performed using its software version 6.8.1.0. The abbreviations for all layers of the automated macular segmentation provided by Spectralis SD-OCT software can be found on the right margin. Abbreviations: ILM, inner limiting membrane; RNFL, retinal nerve fiber layer; GCL, ganglion cell layer; IPL, inner plexiform layer; INL, inner nuclear layer; OPL, outer plexiform layer; ELM, external limiting membrane; PR1, photoreceptor inner segments; PR2, photoreceptor outer segments; RPE, retinal pigment epithelium; BM, Bruch membrane.

The Spectralis SD-OCT software version 6.8.1.0. automatically segments the retina into the IRL and ORL. IRL extends from the inner limiting membrane (ILM) to the external limiting membrane (ELM), whereas ORL extends from the ELM to the Bruch membrane (BM). It further divides the IRL into 6 layers, the RNFL, the ganglion cell layer (GCL), the inner plexiform layer (IPL), the inner nuclear layer (INL), the outer plexiform layer (OPL), and the outer nuclear layer (ONL), as shown in Figure 2.

### 2.3. Statistical Analysis

Data analysis was conducted by E.O.-H. using the Statistical Package for the Social Sciences Software (SPSS version 25, SPSS Inc., IBM Corporation, Armonk, NY, USA). Demographic variables and clinical characteristics were analyzed descriptively and through frequency-based analysis. Values were assessed for normal distribution using the Kolmogorov–Smirnov test. Subsequently, the paired samples *t*-test for parametric samples was performed to investigate differences between the two studied time points based on microperimetry and OCT parameters. A *p*-value of less than 0.05 was deemed statistically significant. In addition, a linear regression analysis was performed to examine the relationship between BCVA and macular integrity. Scatterplots with regression lines were used to visualize this relationship, and violin plots were generated to visualize the distribution and density of the data.

## 3. Results

### 3.1. Demographics

In 2023, the mean age of the DM1 patients was 43.19 ± 10.17 years (range 24–65), and the duration of the disease was 29.84 ± 8.98 years (range 18–48), with moderate or good glycemic control (HbA1c: 7.55 ± 0.90%). Regarding gender, 14 patients were women (43.75%), and 18 were men (56.25%). Systemic parameters of diabetic disease, such as lipid levels, are shown in Table 1, with no significant differences noted between the two time points.

In 2018, DM1 patients presented a BCVA of −0.14 ± 0.12 LogMAR, measured with the 100% contrast ETDRS test; IOP of 17.31 ± 2.68 mmHg; AL of 23.83 ± 1.16 mm; and SE of −0.64 ± 1.68 D. In 2023, patients presented a BCVA of 0.03 ± 0.07 LogMAR, IOP of 16.88 ± 1.86 mmHg, AL of 23.72 ± 1.11 mm, and SE of −0.77 ± 1.69 D. Significant differences were found only in the BCVA between 2018 and 2023, with *p* < 0.001 (Table 2), which means worsening in the BCVA that could be related to disease progression and aging.

### 3.2. Macular Sensitivity Results and Relation with BCVA

**Figure 3:**
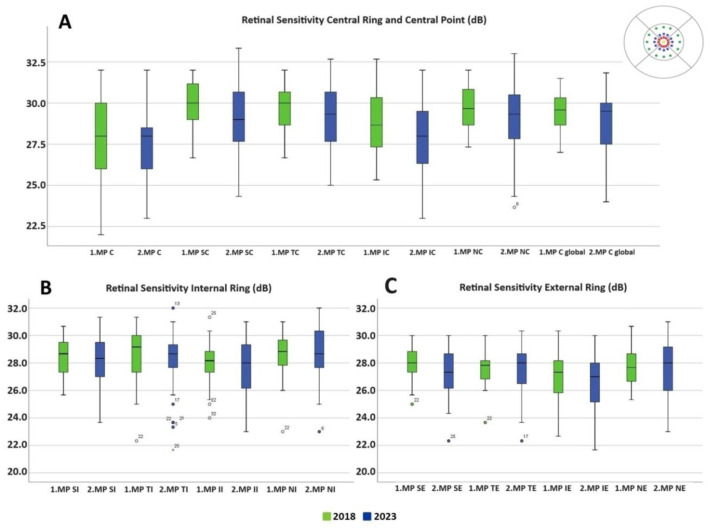
Retinal sensitivity in dB measured by the MAIA microperimeter in different MAIA areas represented in box plots. (**A**) Central ring and central point. (**B**) Internal ring. (**C**) External ring. The 2018 values are shown in green and 2023 values in blue. No values reached statistical significance. Abbreviations: MP: microperimetry; SE: superior external; TE: temporal external; IE: inferior external; NE: nasal external; SI: superior internal; TI: temporal internal; II: inferior internal; NI: nasal internal; C: central; SC: superior central; TC: temporal central; IC: inferior central; NC: nasal central; C Global: central global.

There were no differences in macular sensitivity in any of the macular rings between 2018 and 2023 (Table 3 and Figure 3). The mean of the central macular sensitivity value was 27.56 ± 2.50 dB in 2018 compared to 27.50 ± 2.49 dB in 2023 with *p* = 0.893. Macular integrity increased from 28.8 ± 25.58 to 42.64 ± 34.37 from 2018 to 2023 with *p* = 0.045. Fixation stability P1 and P2 remained unchanged (Table 4). Although no significant differences were found between 2018 and 2023 in the different MAIA areas, Figure 3 illustrates how values in both the central ring and the inner and outer rings (inferior and nasal areas) are more dispersed in 2023.

**Figure 4:**
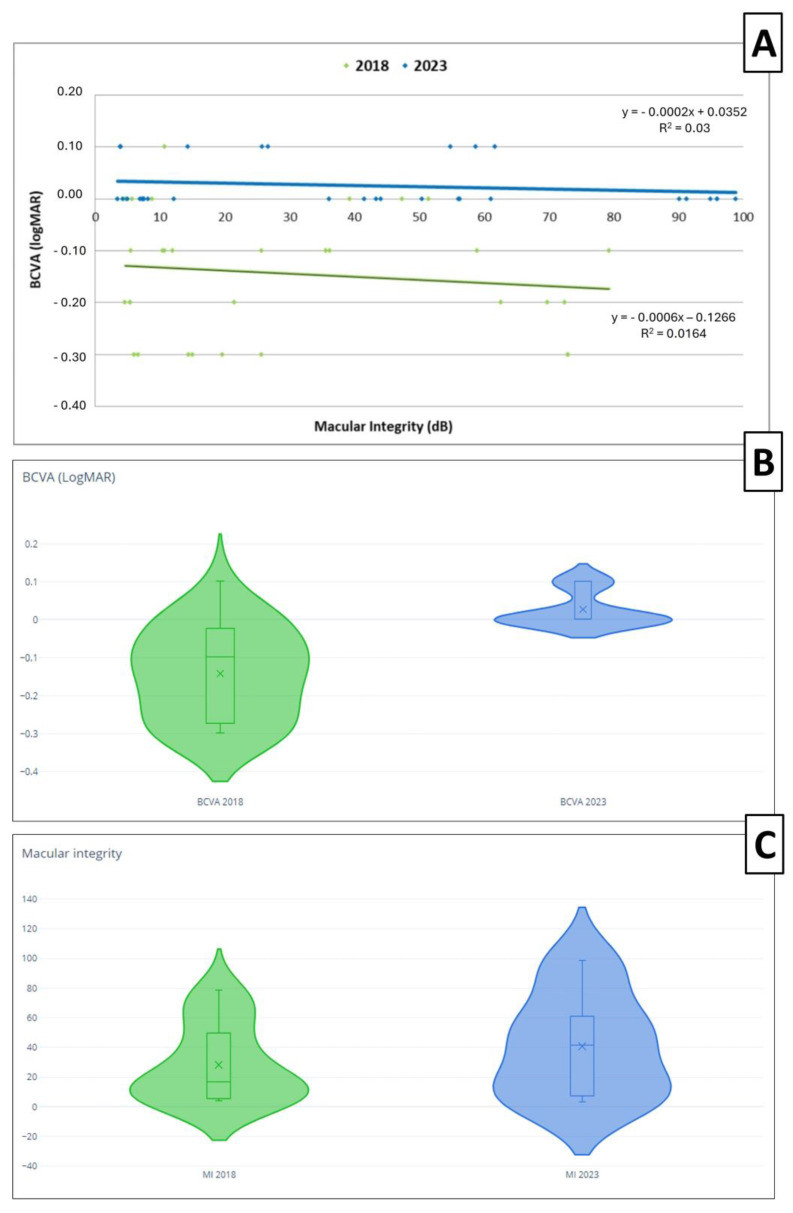
(**A**) Scatter plot and regression lines of the best corrected visual acuity (BCVA) and macular integrity in 2018 (green) and 2023 (blue). The formula of the line at both time points are presented in the figure. (**B**) The violin plot representation of the BCVA in 2018 (green) and 2023 (blue). (**C**) The violin plot representation of macular integrity (MI) in 2018 (green) and 2023 (blue).

Upon analyzing the relationship between the BCVA and macular integrity, we observed a better BCVA and macular integrity in 2018 compared to 2023. As depicted in the scatter plot and violin plots (Figure 4), the BCVA obtained in 2018 was generally better, with the majority being below 0 on the LogMAR scale, while in 2023, the BCVA tended to be 0 or higher. Regarding macular integrity, the worst macular integrity worsened from 80 dB to 100 dB from 2018 to 2023 (Figure 4). There was a negative correlation without statistical significance between the BCVA and macular integrity when their relationship was analyzed and represented with regression lines. The correlation coefficients (r) were r = −0.128 with *p* = 0.484 and a 95% confidence interval of −0.432 to 0.181 and r = −0.173 with *p* = 0.352 and of 95% confidence interval −0.425 to 0.134 in 2018 and in 2023, respectively.

### 3.3. OCT Thickness Analysis

**Figure 5:**
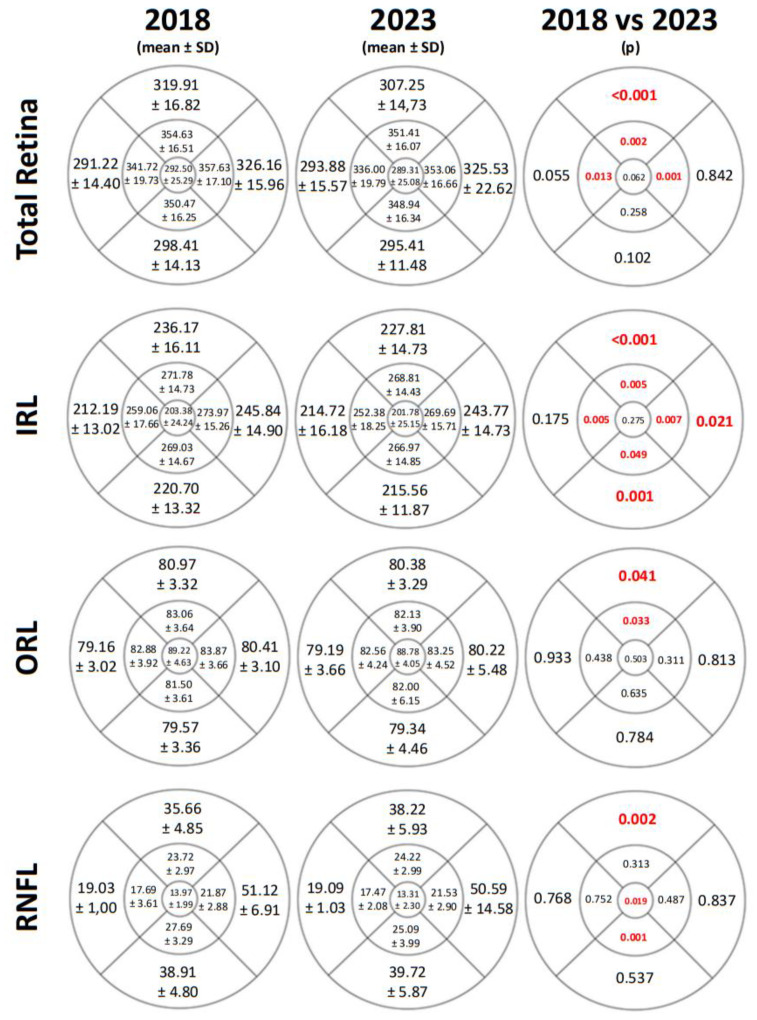
The mean, standard deviation (SD), and *p*-values of total retinal thickness, inner retinal layers (IRL) thickness, outer retinal layers (ORL) thickness, and retinal nerve fiber layer (RNFL) thickness in μm measured by Spectralis SD-OCT and represented in the ETDRS grids from a right eye and in 2018 and in 2023. The *p*-values of less than 0.05 were deemed statistically significant and are shown in red and bold.

We observed differences between the two time points in TR thickness, IRL, ORL, and RNFL in some ETDRS areas measured by SD-OCT. Changes were noted in all areas of the IRL with *p* < 0.05 except for the central area and the outer temporal (OT) quadrant. However, alterations in RNFL thickness were only evident in some of the vertical areas. The TR thickness was also reduced, particularly in the parafoveal and superior areas. All thickness values and *p*-values are shown is Figure 5.

## 4. Discussion

Functional changes are present in DM patients even prior to the onset of the DR, including a reduction in the BCVA and alterations in other functional parameters, such as decreased contrast sensitivity (CS) [22,23], changes in the amplitude or implicit time of full-field or multifocal electroretinogram (ERG) waves [24,25], or reductions in RS with disease progression, as reported by other authors [5,6,26,27].

Our final group comprised 32 eyes of 32 long-term DM1 patients without DR, and we compared changes in macular sensitivity and retinal thickness between 2018 and 2023. We lost some patients from the 2018 cohort due to various reasons, mainly the development of DR lesions, contributing to the small sample size. It is challenging to maintain follow-up with DM1 patients over an extended period without the development of vascular signs or other associated conditions.

We found significant changes between both time points in the BCVA, but we did not observe changes in the systemic parameters. The significant worsening of the BCVA highlights a potential link between disease progression and aging, emphasizing the need for ongoing visual monitoring in long-term DM1 patients, even when DR is not clinically evident. Moreover, we were unable to identify significant changes in RS after 5 years. One reason could be the adequate glycemic control, which remained consistent between the two time points, but the reduction could be also related to aging [28,29]. Sacconi at al. studied a group of 12 well-controlled, long-term (>30 years) DM1 patients without DR signs in 2019 and 2020 and did not find significant changes in RS measured with microperimetry compared to a group of healthy subjects [30,31].

We did not observe changes in any of the parameters related to fixation, including fixation loss, fixation stability, and fixation plot. Gaze fixation serves as a useful tool and is complementary to RS. However, RS, unlike gaze fixation, is contingent on the visual pathway. Several studies have demonstrated that these two aspects are complementary and utilizing them together can enhance the efficacy of microperimetry as a screening test for type 2 diabetes mellitus (DM2) patients with cognitive impairment. Additionally, this combined approach proves valuable in monitoring these patients over the years [16,32]. Fixation parameters have also been studied with microperimetry in pathologies such as glaucoma, revealing a relationship between open-angle glaucoma and a deterioration in the fixation pattern, which worsens as the pathology progresses [33]. Although no changes were observed in the fixation parameters, a significant increase in the macular integrity index was identified after a 5-year follow-up, indicating a decrease in macular function by 2023. Other authors have reported worse macular integrity in DM patients compared to healthy subjects [34] or in patients with diabetic macular edema (DME), where the deterioration in macular integrity is pronounced in patients with severe DME [35].

If we analyze the BCVA and macular integrity, significant differences were found between 2018 and 2023. We observed a better BCVA and macular integrity in 2018 compared to 2023. This also could be attributed to patients experiencing a decline in their condition over time. Other authors have reported poorer macular integrity in a group of DM2 patients compared to healthy subjects, where diabetic patients exhibited compromised macular integrity and lower BCVA values [36].

Other neurodegenerative diseases, not only retinal but also neurological, could also affect RS, including glaucoma, Alzheimer’s disease (AD), age-related macular degeneration (AMD), Stargardt disease, and aging. [9,28,37,38,39]. Cognitive impairment is a crucial factor to consider during microperimetry evaluations, as it may impact RS. Studies by Ortiz et al. [16] and Ciudin et al. [39,40] have identified a correlation between DM2 and cognitive impairment, AD, and dementia. They concluded that measuring RS through microperimetry is a valuable tool, highlighting DM2 as both an accelerator and risk factor for cognitive decline or AD. Additionally, other researchers have also found changes in cognitive dysfunction and its expedited progression associated with DM1 [41].

Beyond ocular pathologies, other factors like pupil size, repeated measurements, and the learning effect during microperimetry can influence RS results obtained via the microperimeter [42,43]. Pupil size may also impact sensitivity results obtained by the MAIA microperimeter, which can be attributed to differences in the amount of light reaching the retina. In our study, all MAIA measurements were conducted without pupil dilatation and under scotopic conditions. Han et al. [42] investigated the effects of pupil dilatation and microperimetry in both healthy individuals and those with retinal diseases in 2017, finding no significant differences between groups [42]. Other studies, such as Lindenmuth et al. [44], Kudrna at al. [45], and Park et al. [46], have revealed statistically significant decreases in threshold sensitivities when looking for the impact of pupil dilation on automated perimetry.

Another notable consideration is the presence of fluctuations in RS or a learning effect in repeated microperimetry measurements by the same patient. Previous studies have shown that eyes with macular diseases such as AMD may exhibit higher sensitivity fluctuations across repeated microperimetric tests [43,47]. In our study, patients underwent MAIA evaluations at two time points, with no intermediate measurements.

Regarding OCT results, we observed significant changes in most of the retinal layers. There was a decrease in TR thickness in the superior and both inner horizontal areas. However, the most substantial thickness reduction occurred in the IRL. In 2023, a significant decrease in the IRL thickness was noted compared to 2018 in all ETDRS areas except in the OT quadrant (*p* = 0.175) and in the central area (*p* = 0.275). Authors such as Pinilla et al. [8], Gundogan et al. [48], and Ambiya et al. [49] have also reported a decrease in IRL during the progression of the disease in DM1 patients. The thinning of the ganglion cell complex (GCC) in DM patients has been described in other studies [34,36,50]. Several authors have observed significant thinning in GCL and IPL in patients with DM [36,51] or a reduction in GCL thickness in advanced stages of DR in DM1 patients [52]. This diminution in the GCC has mainly been attributed to a loss of ganglion cells occurring prior to the thinning of the RNFL, with axonal involvement occurring later in time [53].

The differences between 2018 and 2023 were less pronounced in the ORL and RNFL, with significant differences observed solely in the OS and IS quadrants of ORL and in the OS, in the central, and in the inner inferior (II) quadrants of RNFL. Other studies have reported results consistent with our findings, such as a significant decrease in RNFL thickness in a cohort of DM2 patients without DR [54], in DM2 patients with moderate DR but no DME [36], and in the early stages of DM1 [52] and a reduction in RNFL thickness with the progression of DR [55,56]. However, Montesano et al. [51] did not find differences in RNFL and ORL thickness.

Although we observed changes in the IRL in almost all the ETDRS areas, we noticed a retinal thickness reduction primarily affecting the superior retina. Animal models of DR have demonstrated a higher susceptibility to lesions in the superior area of the retina compared to the inferior region, with an increase in the number of microaneurysms [57]. This regional variability in retinal thinning, particularly the more pronounced reduction in the superior retina, may suggest an inherent vulnerability of this area to diabetic damage. The observed thinning could reflect differences in retinal blood flow, oxygenation, or metabolic demands that make the superior retina more susceptible to microvascular complications and neurodegenerative changes. These findings highlight the importance of monitoring specific retinal regions when assessing early diabetic retinal neurodegeneration, as the superior retina may serve as an early indicator of disease progression.

This study is distinguished from previous research by its longitudinal evaluation of RS and retinal thickness in a cohort of DM1 patients who exhibited no signs of DR at baseline. Although the above-mentioned studies have documented anatomical and functional changes in DM1 patients, the extended follow-up over 5 years without clinical DR provides a unique perspective on the evolution of these parameters, specifically in those of the RS. This approach enabled us to detect subclinical changes in the retina that could serve as early indicators of DR development. The innovative aspect of our study lies in its ability to provide long-term data on disease progression at an early stage without apparent clinical manifestations, significantly contributing to the current understanding of DR pathogenesis.

Our study limitation is the small sample of examined individuals, which resulted from the loss of follow-up related over the extended study period. To confirm these findings, further research with a larger cohort of diabetic patients and an extended follow-up period is needed. Exploring DM patients across various stages of DR and comparing the results would also be valuable and necessary, as different stages of the disease may manifest distinct behaviors.

## 5. Conclusions

Long-term DM1 patients without DR lesions exhibited no changes in macular sensitivity after a well-controlled 5-year follow-up. However, these patients demonstrated deteriorated macular integrity and a worse BCVA in 2023. These functional findings are associated with a reduction in retinal layer thickness, primarily affecting the IRL. Unlike other studies, this work conducts a 5-year follow-up and quantifies the changes in macular sensitivity by microperimetry and retinal thickness between both time points. The novelty of our approach lies in the ability to identify subclinical alterations before the onset of evident DR. Future studies with larger sample sizes and longer follow-up periods are necessary to assess and study the changes that diabetic patients undergo as the pathology progresses, which will allow for a better understanding and the preventive management of DR in each stage.

## Figures and Tables

**Table 1:** The mean and standard deviation (SD) of the systemic parameters of diabetic disease in the DM1 patients at the two time points. Abbreviations: DM1, type 1 diabetes mellitus; HbA1c (%), glycosylated hemoglobin; HDL, high-density lipoprotein; LDL, low-density lipoprotein.

	2018	2023	
DM1 Patients	Mean ± SD	Mean ± SD	*p*-Value
HbA1c (%)	7.59 ± 1.01	7.55 ± 0.90	0.420
Creatinine (mg/dL)	0.80 ± 0.12	0.77 ± 0.12	0.382
Total Cholesterol (mg/dL)	194.50 ± 35.63	195.80 ± 32.35	0.355
HDL Cholesterol (mg/dL)	57.04 ± 14.04	57.57 ± 13.55	0.411
LDL Cholesterol (mg/dL)	120.21 ± 27.65	121.63 ± 24.82	0.340
Microalbumin in Urine (mg)	9.04 ± 7.85	6.78 ± 7.20	0.476

**Table 2:** The mean and standard deviation (SD) in the two time points of the type 1 diabetic mellitus (DM1) patients of the best corrected visual acuity (BCVA) in the LogMAR scale, spherical equivalent (SE) in diopters (D), axial length (AL) in mm, and intraocular pressure (IOP) in mmHg. Statistically significant differences (*p* < 0.05) are in bold and red.

	2018	2023	
DM1 Patients	Mean ± SD	Mean ± SD	*p*-Value
BCVA (LogMAR)	−0.14 ± 0.12	0.03 ± 0.07	**<0.001**
SE (D)	−0.77 ± 1.69	−0.64 ± 1.68	0.179
AL (mm)	23.72 ± 1.11	23.83 ± 1.16	0.226
IOP (mmHg)	17.31 ± 2.68	16.88 ± 1.86	0.174

**Table 3:** The mean, standard deviation (SD), and statistical significance (*p*-value) of retinal sensitivity in dB measured by the MAIA microperimeter in the different areas. Abbreviations: SE: superior external; TE: temporal external; IE: inferior external; NE: nasal external; SI: superior internal; TI: temporal internal; II: inferior internal; NI: nasal internal; C: central; SC: superior central; TC: temporal central; IC: inferior central; NC: nasal central; C Global: central global.

	2018	2023	
MAIA Areas	Mean (dB) ± SD	Mean (dB) ± SD	*p*-Value
SE	27.94 ± 1.13	27.25 ± 1.87	0.072
TE	27.61 ± 1.24	27.23 ± 2.03	0.340
IE	26.96 ± 1.83	26.36 ± 2.19	0.129
NE	27.88 ± 1.32	27.45 ± 2.34	0.289
SI	28.39 ± 1.39	28.23 ± 1.84	0.697
TI	28.44 ± 1.91	28.03 ± 2.37	0.384
II	28.17 ± 1.64	27.64 ± 2.19	0.244
NI	28.56 ± 1.61	28.67 ± 2.15	0.810
C	27.56 ± 2.50	27.50 ± 2.49	0.893
SC	29.68 ± 1.60	28.94 ± 2.12	0.062
TC	29.40 ± 1.86	29.07 ± 2.02	0.415
IC	28.63 ± 1.97	27.90 ± 2.36	0.078
NC	29.62 ± 1.49	28.86 ± 2.35	0.121
C Global	29.32 ± 1.35	28.73 ± 1.93	0.079

**Table 4:** The mean and standard deviation (SD) of MAIA parameters comparing 2018 and 2023. The macular integrity and average threshold are measured in dB, the BCEA angle is measured in degrees, the area is measured in square degrees and fixation loses, and P1, P2 are reported in %. Abbreviations: BCEA, bivariate contour ellipse angle. Statistically significant differences (*p* < 0.05) are in red and bold.

	2018	2023	
MAIA Parameter	Mean ± SD	Mean ± SD	*p*-Value
Macular integrity (dB)	28.80 ± 25.58	42.64 ± 34.37	**0.045**
Average threshold (dB)	28.43 ± 1.27	27.99 ± 1.87	0.168
Fixation stability P1 (%)	90.97 ± 14.07	97.63 ± 3.31	0.313
Fixation stability P2 (%)	97.84 ± 4.67	97.63 ± 3.31	0.815
BCEA 63 area (°^2^)	1.18 ± 1.41	1.19 ± 1.20	0.965
BCEA 63 angle (°)	−7.10 ± 66.36	14.60 ± 63.49	0.198
BCEA 95 area (°^2^)	2.53 ± 7.03	3.56 ± 3.61	0.408
BCEA 95 angle (°)	−7.87 ± 66.76	14.60 ± 63.49	0.184
Fixation loses (%)	2.03 ± 6.46	4.53 ± 9.62	0.208

## Data Availability

The data presented in this study are available within the article.
